# Microstructure Design of Tempered Martensite by Atomistically Informed Full-Field Simulation: From Quenching to Fracture

**DOI:** 10.3390/ma9080673

**Published:** 2016-08-09

**Authors:** Efim Borukhovich, Guanxing Du, Matthias Stratmann, Martin Boeff, Oleg Shchyglo, Alexander Hartmaier, Ingo Steinbach

**Affiliations:** 1 Interdisciplinary Centre for Advanced Materials Simulations (ICAMS), Ruhr-Universität Bochum, Universitätsstr. 150, Bochum 44801, Germany; guanxing.du@rub.de (G.D.); matthias.stratmann@rub.de (M.S.); martin.boeff@rub.de (M.B.); oleg.shchyglo@rub.de (O.S.); alexander.hartmaier@rub.de (A.H.); ingo.steinbach@rub.de (I.S.); 2 Department of Materials Science and Engineering, KTH Royal Institute of Technology, Stockholm 10044, Sweden

**Keywords:** phase-field, martensite, quenching, fracture, finite strain

## Abstract

Martensitic steels form a material class with a versatile range of properties that can be selected by varying the processing chain. In order to study and design the desired processing with the minimal experimental effort, modeling tools are required. In this work, a full processing cycle from quenching over tempering to mechanical testing is simulated with a single modeling framework that combines the features of the phase-field method and a coupled chemo-mechanical approach. In order to perform the mechanical testing, the mechanical part is extended to the large deformations case and coupled to crystal plasticity and a linear damage model. The quenching process is governed by the austenite-martensite transformation. In the tempering step, carbon segregation to the grain boundaries and the resulting cementite formation occur. During mechanical testing, the obtained material sample undergoes a large deformation that leads to local failure. The initial formation of the damage zones is observed to happen next to the carbides, while the final damage morphology follows the martensite microstructure. This multi-scale approach can be applied to design optimal microstructures dependent on processing and materials composition.

## 1. Introduction

Martensitic transformation is a diffusionless solid state phase transformation happening at some critical temperatures by applying a rapid cooling to austenite. Martensite is considered to be the backbone of many high-strength commercial steels. Therefore, for more than a century, an ample amount of experimental work has been done to understand the crystallographic features of martensitic transformation by using optical microscopy and, recently, more accurate techniques based on scanning electron microscopy (SEM) [1].

Previous multi-scale studies on martensite, which took the effect of precipitate networks into account [2], focused on the effect of M23C6 carbides in a P91 creep resisting steel. In contrast, this work deals with the effect on M3C carbide (cementite) growth on the mechanical properties of a quenched and tempered steel, as well as the effect of carbon depletion in the martensitic matrix. Furthermore, the microstructure is not randomly generated, but simulated beginning with a martensitic transformation and followed by a precipitation step.

From the modeling point of view, the current work is based on three components. The core of the used modeling framework OpenPhase [3] is the multi-phase-field method (MPF) developed and presented in [4,5,6]. Its main purpose and at the same time its strength is the ability to describe phase transformation processes caused and accompanied by the effects of thermodynamics and mechanics and considering these. A short description of the MPF method follows in Section 2.

An important breakthrough for the phase-field community was the coupling between phase-field and linear elastic models, e.g., [7]. In the last few years, an extension of the linear mechanical framework to the case of large deformations was published [8,9]. In Section 3, both mechanical limits are presented. Furthermore, an extension of the large deformations framework by a crystal plasticity law and a linear damage model are given.

The third and last component is the thermodynamic model. The diffusion model used for the presented calculations is the finite interface dissipation model published in [10,11]. A short introduction to it together with mechanical coupling as formulated in [12,13] is given in Section 4.

The result part is divided into three parts. First, the quenching process is modeled (Section 5). During this process, the austenitic steel transforms into martensite due to the temperature drop. Since this transformation is governed by the minimization of the strain energy, the different martensite variants will be selected automatically to construct the hierarchical microstructures consisting of the so-called packets, blocks and sub-blocks. In the second result part, in Section 6, the tempering process is modeled. This process is governed by carbon segregation to the grain boundaries, which leads to carbon oversaturation, leading to the nucleation and growth of carbides. Finally, as the last results part, a mechanical test with 10% uniaxial strain of the sample is presented in Section 7. Due to the high stiffness contrast between the carbides and the martensitic matrix, stress maxima leading to failure evolve in the corresponding grain boundary regions. Once the damage has nucleated, it evolves rapidly through the corresponding martensite grains, leading to a damage distribution following the martensite morphology.

## 2. Multi-Phase Field Method

Martensite is a complex microstructure, which contains multiple variants with different transformation strains and crystallographic orientations. Therefore, in order to simulate martensitic transformation and martensitic structures, the multi-phase field (MPF) model is employed to describe the interaction between different martensite variants in this work.

The concept of the MPF model was proposed by Steinbach et al. [4,5,6]. Different phases are represented by the indicator functions, which are also called phase field variables $\phi_{\alpha }\left(x,t\right)$ in the MPF model. For an *N* phases system, the phase-field variables $\phi_{\alpha }$ are constrained according to:

(1) $$\sum_{\alpha =1}^{N}\phi_{\alpha }=1$$

where $\phi_{\alpha }\in \left[0,1\right]$ and $\phi_{\alpha }=0$ indicates a non-existing phase.

The MPF model starts from a general description of the total free energy as an integral of the energy density functional over the domain Ω [14]. For the martensitic transformation, the free energy functional is split into three parts: the interfacial energy density $f^{intf}$, the chemical energy density $f^{ch}$ and the elastic energy density $f^{el}$:

(2) $$F=\int_{\Omega }\left(f^{intf}+f^{ch}+f^{el}\right)$$

The interfacial energy density between all pairs of *N* phases is given by [14]:

(3) $$f^{intf}=\sum_{\alpha =1}^{N}\sum_{\beta \neq \alpha ,\beta =1}^{N}\frac{4\gamma_{\alpha \beta }}{\eta }\left\{-\frac{\eta^{2}}{\pi^{2}}\nabla \phi_{\alpha }\cdot \nabla \phi_{\beta }+\left|\phi_{\alpha }\phi_{\beta }\right|\right\}$$

where $\gamma_{\alpha \beta }$ is the interfacial energy between the phases *α* and *β* and *η* is the interface width. Here, the interface width *η* is a global numerical parameter, which should be larger than the atomic distance and smaller than the typical scale of the modeled microstructure. In order to quantitatively capture the physics of interest, the MPF model has been formulated to be independent of *η*.

In the MPF model, the time evolution equation can be derived from:

(4) $$\dot{\phi_{\alpha }}=-\sum_{\beta =1}^{N}\frac{\mu_{\alpha \beta }}{N}\left(\frac{\delta F}{\delta \phi_{\alpha }}-\frac{\delta F}{\delta \phi_{\beta }}\right)$$

where $\mu_{\alpha \beta }$ is the interface mobility for each pair of phases. Inserting the free energy densities, the multi-phase field equation can be computed and generalized by using a double obstacle potential. For more details on the MPF model, see [14,15,16].

In order to simulate the martensitic transformation and microstructure evolution in low carbon steels, in this work, the specific solutions for the transformation thermodynamics and mechanics, as well as the precipitation process were introduced into the general MPF model. They are discussed in the following sections.

## 3. Mechanics

### 3.1. Linear Limit

The finite strain description used in the current work and presented in the next section is based on a linear small strain description that considers the effect of phase transformation. Its core is an iterative spectral solution of the equilibrium of the mechanical stress ***σ*** condition (for formulation details, see [7]):

(5) ∇·σ=0

In short, the method assumes the crystal lattice being distorted by a phase transformation, resulting in a spatially-dependent eigenstrain $\epsilon^{*}$ and adjusts the global total strain solution to fulfill Equation (5), while the eigenstrain enters the stress field through a modified Hooke’s law:

(6) $$\sigma =C(\epsilon -\epsilon^{*})$$

The coupling with the phase-field model introduced above occurs through the definition of the stiffness and eigenstrain tensors $C_{\alpha }$ and $\epsilon_{\alpha }^{*}$ for each phase $\phi_{\alpha }$ individually. To obtain effective stiffness and eigenstrain values in volume elements with multiple phases, homogenization assumptions are to be made. Typical choices are the iso-stress and the iso-strain assumptions. These postulate that the deformation results in either equal stress or in equal strain in the two phases. The resulting homogenization for eigenstrain in both cases is:

(7) $$\begin{array}{c} \epsilon^{*}=\sum_{\alpha }\phi_{\alpha }\epsilon_{\alpha }^{*} \end{array}$$

while for stiffness, either linear averaging of stiffness or linear averaging of compliance ($C^{-1}$) arises ([17]):

(8) $$\begin{array}{ccc} C_{iso-stress} & = & \sum_{\alpha }\phi_{\alpha }C_{\alpha } \end{array}$$

(9) $$\begin{array}{ccc} C_{iso-strain} & = & {\left(\sum_{\alpha }\phi_{\alpha }C_{\alpha }^{-1}\right)}^{-1} \end{array}$$

In the current work, the iso-stress assumption is made. Due to the phase field dependency of Equations (7) and (8) according to Equation (4) and assuming that stress is constant during transformation, a mechanical contribution to the driving force follows:

(10) $$\begin{array}{c} dG_{\alpha \beta }^{mech}=\frac{\delta F^{mech}}{\delta \phi_{\alpha }}-\frac{\delta F^{mech}}{\delta \phi_{\beta }}=\sigma \left(\epsilon_{\alpha }^{*}-\epsilon_{\beta }^{*}\right)+\frac{1}{2}\sigma \left(C_{\alpha }^{-1}-C_{\beta }^{-1}\right)\sigma \end{array}$$

using the mechanical energy:

$$\begin{array}{c} F^{mech}=\frac{1}{2}\left(\epsilon -\epsilon^{*}\right)C\left(\epsilon -\epsilon^{*}\right) \end{array}$$

### 3.2. Large Deformation Framework

In order to describe deformation processes, two aspects are to be considered, the geometry evolution and the corresponding energy evolution. The first can be uniquely given by the displacement u, the second by the (Cauchy) stress ***σ***. In small deformation approaches, the first can be neglected, after its effect on stress has been taken into account. However, in order to consider the influence of the geometrical non-linearity on the material processes and behavior, it must not be dismissed.

Starting with the implicit definition of the displacement of a material point with the coordinates X in the non-deformed configuration and x being the coordinates in the deformed configuration:

(11) $$\begin{array}{c} x=X+u \end{array}$$

it is convenient to define the deformation gradient tensor F:

(12) $$\begin{array}{c} F=\frac{\partial x}{\partial X}=1+\frac{\partial u}{\partial X} \end{array}$$

and to apply the polar decomposition:

(13) $$\begin{array}{c} F=RU \end{array}$$

that splits the energetically-neutral skew symmetric rotational deformation part R from the symmetric stretch part U. In order to obtain the two tensors, we have to calculate the product:

(14) $$\begin{array}{c} U^{2}=F^{T}F \end{array}$$

Assuming that the deformation process is solved incrementally, with increments small enough to assume $\frac{\partial u}{\partial X}\approx \frac{\partial u}{\partial x}$ and to neglect its second order terms, we obtain:

(15) $$\begin{array}{c} U^{2}={\left(1+\frac{\partial u}{\partial X}\right)}^{T}\left(1+\frac{\partial u}{\partial X}\right)\approx 1+\frac{\partial u}{\partial x}+{\frac{\partial u}{\partial x}}^{T} \end{array}$$

and thus, the final approximation for U results in (here, we use that the single application of the operator $U^{2}$ corresponds to applying the operator U twice and the assumption that $U^{2}\approx 1$):

(16) $$\begin{array}{c} U_{approx}=1+\frac{1}{2}\left(\frac{\partial u}{\partial x}+{\frac{\partial u}{\partial x}}^{T}\right) \end{array}$$

In fact, Equation (16) is strongly related to the engineering strain $\epsilon_{eng}=\frac{1}{2}\left(\frac{\partial u}{\partial x}+{\frac{\partial u}{\partial x}}^{T}\right)\approx U_{approx}-1$. This insight is not surprising, since we had to make the assumption of small strain to obtain the above stretch approximation. However, integrating the engineering strain would throw us back to a usual small strain formulation. Therefore, the small strain limitation is only assumed for the deformation increments. The total deformation is calculated via the integration of the logarithmic strain increment:

(17) $$\begin{array}{c} \Delta \epsilon_{ln}=ln\left(U_{approx}\right) \end{array}$$

that is additive, contrary to the engineering strain. It should be noted that the logarithm of the stretch tensor has to be calculated with the usual tensorial logarithm function:

(18) $$\begin{array}{c} ln\left(\tau \right)=\sum_{i}ln\left(\lambda_{i}\right)n_{i}⊗n_{i} \end{array}$$

where $\lambda_{i}$ and $n_{i}$ are the corresponding eigenvalues and eigenvectors of ***τ*** ([18]).

In the same spirit, we continue to obtain the rotational contribution to the deformation. We start with:

(19) $$\begin{array}{c} R^{2}=FF^{-T} \end{array}$$

where $\tau^{-T}$ stands for the transposed inverse of a tensor. Neglecting the second order terms yields:

(20) $$\begin{array}{c} R^{2}=\left(1+\frac{\partial u}{\partial x}\right)\left(1-{\frac{\partial u}{\partial x}}^{T}\right)\approx 1+\frac{\partial u}{\partial x}-{\frac{\partial u}{\partial x}}^{T} \end{array}$$

Analogue to (16), we obtain the approximated rotation tensor by:

(21) $$\begin{array}{c} R_{approx}=1+\frac{1}{2}\left(\frac{\partial u}{\partial x}-{\frac{\partial u}{\partial x}}^{T}\right) \end{array}$$

The approximated rotation tensor can be related to the rotation increment tensor:

(22) $$\begin{array}{c} \Delta R=\frac{1}{2}\left(\frac{\partial u}{\partial x}-{\frac{\partial u}{\partial x}}^{T}\right) \end{array}$$

In order to obtain the rotation tensor representing the rotation part of the total large deformation, the rotation increments are accumulated via Rodrigues’ formula.

Having the proper variables to describe the geometry evolution, the logarithmic strain and the rotation tensors, the influence of the geometrical non-linearity has to be considered in the stress calculation. In the small deformation approaches, the linear Hooke’s law is used, which relates the strain and the stress in a linear manner, with the stiffness tensor C as the linear coefficient. Due to the incremental approach followed in the current work, the linear Hooke’s law is kept for the stress description of the stress increments Δσ in each small deformation step. The stress integration occurs via linear addition and consideration of the geometry change due to rotation:

(23) $$\begin{array}{c} \sigma^{n+1}=\Delta R\sigma^{n}\Delta R^{T}+\Delta \sigma \end{array}$$

with the superscript *n* and n+1 denoting the previous and current deformation steps. Finally, in order to apply the translational part of the deformation, the stress is advected using the compressible advection equation:

(24) $$\begin{array}{c} \dot{\sigma }=-\nabla \cdot \left(v⊗\sigma \right) \end{array}$$

where v is the velocity vector resulting from the displacement:

(25) $$\begin{array}{c} v=\frac{\Delta u}{\Delta t} \end{array}$$

The geometry evolution is considered in the case of the other non-mechanical physical variables in a similar way. Anisotropic, tensorial variables (of rank two and four) are rotated:

(26) $$\begin{array}{ccc} \tau_{2}^{n+1} & = & \Delta R\tau_{2}^{n}\Delta R^{T}, \end{array}$$

(27) $$\begin{array}{ccc} \tau_{4}^{n+1} & = & \Delta R\Delta R\tau_{4}^{n}\Delta R^{T}\Delta R^{T} \end{array}$$

Conserved variables are advected with the compressible advection equation:

(28) $$\begin{array}{c} {\dot{\tau }}_{cons}=-\nabla \cdot \left(v⊗\tau_{cons}\right) \end{array}$$

while the non-conserved ones (like phase-field or velocity) are advected with the incompressible version (it should be noted, that the tensorial Equations (28) and (29) hold also for scalar variables):

(29) $$\begin{array}{c} {\dot{\tau }}_{cons}=-v⊗\left(\nabla \cdot \tau_{cons}\right) \end{array}$$

Summing up, the used large deformation framework is based on the subdivision of the total deformation into linear deformation steps. The displacement increments Δu are obtained from the stress equilibrium condition for the displacement increment-dependent stress increment:

(30) $$\begin{array}{c} \nabla \cdot \Delta \sigma (\Delta u)=0 \end{array}$$

yielding the logarithmic strain and rotation increments $\Delta \epsilon_{ln}$ and ΔR. Subsequently, the geometry evolution corresponding to the two tensor fields is applied via the advection Equations (28) and (29) and rotation updates Equations (26) and (27) to the system describing variables, especially the stress.

An interested reader can find more deeper description of the used large deformations framework in [8,9,19], where aspects, such as homogenization of mechanical properties, objectivity and further implementation details are discussed, and result comparisons with Abaqus ([20]) are performed. The used iterative solution of Equation (30) is described in [7].

The obtained linearized formulation has the advantage of being straight forward to be implemented and coupled with the explicitly-formulated phase-field model. It makes use of the small time step used in phase transformation modeling and omits the need for complex mesh geometry working in the Eulerian frame of reference.

### 3.3. Crystal Plasticity Model

A flow rule is provided for the model in Equation (32). Though it neglects strain hardening, it is sufficient to perform mechanical modeling considering the plasticity effects. The stress relaxation due to crystal slip is considered via the crystal plasticity model given by:

(31) $$\begin{array}{ccc} \Delta \epsilon_{pl} & = & \sum_{s}\dot{\gamma_{s}}\Delta tb_{s}⊗n_{s} \end{array}$$

(32) $$\begin{array}{ccc} \dot{\gamma_{s}} & = & {\dot{\gamma }}_{s}^{0}{\left|\frac{\tau_{s}^{rss}}{\tau_{s}^{crss}}\right|}^{k}sgn\left(\tau_{s}^{rss}\right) \end{array}$$

(33) $$\begin{array}{ccc} \tau_{s}^{rss} & = & \sigma :(b_{s}⊗n_{s}) \end{array}$$

The parameters used are the Burgers vector b, the slip plane normal n, the resolved shear stress $\tau^{rss}$, the critical resolved shear stress $\tau^{crss}$, the slip rate coefficient ${\dot{\gamma }}_{s}^{0}$, the slip exponent *k* and the time step Δt. $\Delta \epsilon_{pl}$ denotes the increment in the plastic strain tensor and $\dot{\gamma_{s}}$ the slip rate on the particular slip system, which is defined by $b_{s}⊗n_{s}$. Thus, the physical interpretation of the used model is: given a material with a set of slip systems $b_{s}⊗n_{s}$ and the stress state ***σ***, project the stress on each slip system (Equation (33)), calculate the resulting slip (Equation (32)) and sum the slip contributions for all slip systems to yield the plastic strain increment (Equation (31)).

### 3.4. Damage Model

Based on the accumulated plastic strain in the logarithmic measure (for details, see [21]) given by:

(34) $$p_{n+1}=p_{n}+\Delta p\;\;\text{and}\;\;\Delta p=\ln\left(1+\sqrt{\frac{2}{3}\Delta \epsilon_{pl}:\Delta \epsilon_{pl}}\right)$$

the damage model is evaluated. The increment of plastic equivalent strain is determined by Equation (31). In general, damage models are known to show spurious mesh sensitivity so that they do not converge upon mesh refinement, but produce non-physical results. Following Peerlings et al. [22], to suppress spurious localization, the non-local plastic strain increment $\bar{p}$ is determined by the solution of the Laplace equation:

(35) $$\bar{p}-\alpha \nabla^{2}\bar{p}=p$$

which determines $\bar{p}$ based on a local source term *p* as given by Equation (34) and an internal length scale parameter *α*. At this point, it is stressed that the equation (Equation (35)) is time invariant, thus leading to a time-invariant spatial spread of the local accumulated equivalent plastic strain in the vicinity of the material point considered. Furthermore, a linear dependency of the effective damage *D* on the non-local equivalent plastic strain is assumed. The onset of ductile damage and final failure are defined by a minimum and maximum strain $p_{min}$ and $p_{max}$, respectively, so that:

$$\begin{array}{c} D=\left\{\begin{array}{cc} 0 & \text{for}\;\;\bar{p}<p_{min}\\ \frac{\bar{p}-p_{min}}{p_{max}-p_{min}}\;\;\;\; & \text{for}\;\;p_{min}\leq \bar{p}\leq p_{max}\\ 1 & \text{for}\;\;\bar{p}>p_{max} \end{array}\right. \end{array}$$

**Figure 1:**
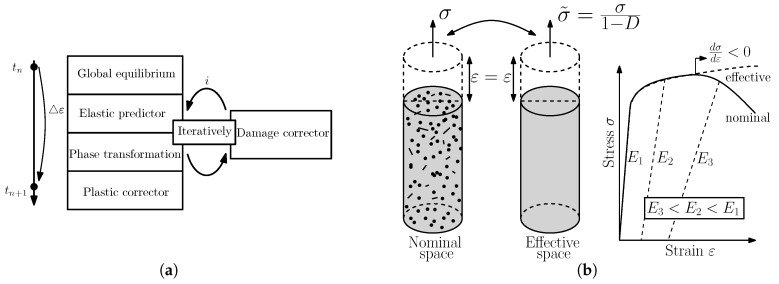
The stress update procedure consists of the global equilibrium, the elastic predictor, the return mapping and the damage correction phase, which are solved subsequently within one iteration for each time step and shown in (**a**); the concept distinguishing between nominal and effective stress is presented in (**b**).

Referring back to the stress increment from the previous Section 3.2, the damage variable connects the stress increment in nominal space to the effective space, which is used in the material model. Following that, it can be written:

(36) $$\Delta \sigma =\left(1-D\right)\Delta \tilde{\sigma }$$

where ***σ*** is the nominal stress and $\tilde{\sigma }$ the effective stress. The concept of nominal and effective space is visualized in Figure 1b and goes back to [23]. While stresses in nominal space consider the evolution of defects in the microstructure and decrease with evolving damage, stresses in the effective space monotonically increase and are considered within the plasticity model.

To solve the boundary value problem, the operator split is used, which is proposed in [24,25,26]. By a discrete time stepping Δt, a corresponding strain increment Δε can be determined, so that the following equation for the stress update procedure can be derived:

(37) $$\begin{array}{ccc} \Delta \sigma_{n+1} & = & \Delta \sigma_{n}+\left(1-D_{n+1}\right)\Delta {\tilde{\sigma }}_{n}-\Delta D\Delta {\tilde{\sigma }}_{n} \end{array}$$

(38) $$\begin{array}{ccc} \Delta D & = & D_{n+1}-D_{n} \end{array}$$

which describes the influence of damage on the stress increment. The operator split is visualized in Figure 1a.

## 4. Thermodynamics and Diffusion

For a multi-component multi-phase field simulation, the description of the phase field model with finite interface dissipation from [11] was carefully chosen, as it allows simulations off-equilibrium, accepts thermodynamic input from thermodynamic databases and is fast enough for a large 3D simulation. In this model, a chemical contribution to the total energy is introduced,

(39) $$\begin{array}{c} f^{chem}=\sum_{\alpha =1}^{N}\phi^{\alpha }f^{\alpha }\left(T,P,{\vec{c}}^{\alpha }\right) \end{array}$$

which creates a chemical driving force:

(40) $$\begin{array}{c} \Delta g^{\alpha \beta }=f^{\beta }-f^{\alpha }-\sum_{i=1}^{n-1}\frac{\phi^{\alpha }{\tilde{\mu }}_{i}^{\alpha }+\phi^{\beta }{\tilde{\mu }}_{i}^{\beta }}{\phi^{\alpha }+\phi^{\beta }}\left(x_{i}^{\beta }-x_{i}^{\alpha }\right) \end{array}$$

The Gibbs energy densities and diffusion potentials were taken from the thermodynamic assessment of the Fe-Cr-Mn-C system from [27] and were written in an analytic equation with the data provided in a thermodynamic database with algorithms provided in [28].

While the thermodynamic data were compiled for phases using the sublattice model [29], a relation was found between sublattice occupancies and the phase composition in mole fractions for the ferritic phase and the cementite, which made more complex extensions to the phase field model and further algorithms unnecessary [30].

**Figure 2:**
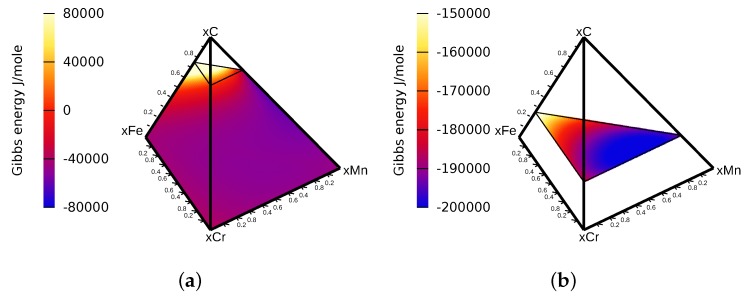
Molar Gibbs energy of (**a**) the ferrite phase and (**b**) the cementite phase.

In Lee’s work [27], the ferrite phase is modeled as a regular solution phase with the sublattice model of (Cr,Fe,Mn)1(C,Va)3, which explains the maximum composition of 75 at% for carbon in Figure 2a. The cementite phase, however, is modeled as a stoichiometric phase with regards to carbon with a fixed composition of 25 at%, so its representation in the component space is a plane with constant carbon composition. To simulate not only stoichiometric phases with the phase field model of finite interface dissipation, extensive modifications have been made to the model and the implementations, to cope with the additional constrains of those phases. Additional modifications had to be made to allow significant composition differences in the interface, as well as a complete depletion of the ferritic phase with certain element compositions.

To extend the BCC model from Lee [27] to the description of the martensitic phase, the additional mechanical energy in the lattice is described in [13]. The energy formulation for a BCTphase therefore includes the chemical energy of the BCCphase and the mechanical energy derived from the tetragonal distortion of the BCT cell.

Diffusion and redistribution of composition are solved within the same framework as described in [11]. Here, the diffusion does not take place on a totally conserved composition field, but on separate composition fields, one for each thermodynamic phase. In the interface, no equilibrium compositions are calculated for each phase, but a redistribution between the phase composition fields happens locally, until the phases reach an equilibrium state over time. Therefore, the composition flux can be split into a diffusion part for each phase composition, a local redistribution part between individual phase compositions and a third part, the kinetic redistribution in the case of phase transformations according to:

(41) $$\begin{array}{ccc} \phi^{\alpha }{\dot{x}}_{i}^{\alpha } & = & v_{M}^{2}\nabla \left(\phi^{\alpha }\sum_{j=1}^{n-1}{\tilde{M}}_{ij}^{\alpha }\nabla {\tilde{\mu }}_{j}^{\alpha }\right)+\sum_{\beta =1}^{N}P_{i}^{\alpha \beta }\frac{\phi^{\alpha }\phi^{\beta }}{\phi^{\alpha }+\phi^{\beta }}\left({\tilde{\mu }}_{i}^{\beta }-{\tilde{\mu }}_{i}^{\alpha }\right)\\ & & +\sum_{\beta =1}^{N}\frac{\phi^{\alpha }}{N\left(\phi^{\alpha }+\phi^{\beta }\right)}{\dot{\Psi }}^{\alpha \beta }\left(x_{i}^{\beta }-x_{i}^{\alpha }\right) \end{array}$$

with the phase composition $x_{i}^{\alpha }$ of phase *α* and component *i*, the molar volume $v_{M}$, the chemical mobility ${\tilde{M}}_{ij}^{\alpha }$, the permeability $P_{i}^{\alpha \beta }$ and the phase fraction increment ${\dot{\Psi }}^{\alpha \beta }$. The total composition of element *i* can be calculated in this case as $x_{i}=\sum_{\alpha =1}^{N}x_{i}^{\alpha }\phi^{\alpha }$. As carbon is the rate-defining element for the growth of cementite precipitates in low alloyed steels, the chemical mobility of chromium and manganese were increased to the same order as carbon with a constant value of $10^{-23}$ $\frac{m^{2}}{J\;s}$. This restriction changes the kinetics of the phase transformation, as it is a diffusion controlled process. Because of this, the final results will not be described by its tempering time, but by its total count of time steps 100,000. It is not given in seconds, but in the number of simulated iterations. The permeability is assumed to be $10^{-6}$ $\frac{m^{3}}{J\;s}$ for chromium and manganese, 0 $\frac{m^{3}}{J\;s}$ for carbon; this is due to the stoichiometric constraint, and carbon is only redistributed with the third part of the diffusion equation.

Mechanical stress is known to influence the diffusion of alloying elements. This behavior on the diffusion was modeled recently by [12]. It uses linear coefficients that quantify the dependency of the transformation strain in each orientation with respect to the alloying composition. This dependency between the composition and the transformation strain, or the lattice parameter of the BCT lattice, was taken from [31]. As the precipitation simulation was simulated only in the elastic regime for simplicity, the values were scaled back accordingly to $\kappa_{1}=-0.005155$, $\kappa_{2}=-0.005155$ and $\kappa_{3}=0.03104$, where $\kappa_{i}$ are the linear expansion coefficients of the elastic tensor on composition.

A similar effect of the alloying composition was recently shown by [32] on the interfacial energy of grain boundaries. This effect can be linearly approximated and modeled in the same way as [12], with the linear expansion coefficient of the interfacial energy on composition $\lambda_{1}=-12$ for carbon.

These two models have a significant effect on the diffusion and the segregation of alloying elements to grain boundaries, which are demonstrated in the chapter (results:precipitations).

## 5. Results: Martensite Microstructure

For simplicity, the chemical composition of model steel is considered as 2 wt% Mn, 1 wt% Cr and 0.1 wt% C. Additionally, the applied heat treatment process is: after austenization at 950 °C, the austenite is quenched to 0 °C with a fixed cooling rate of 100 °C/s; then, the transformed martensite is held at 0 °C for an additional six seconds. Since the common martensitic transformation is diffusionless and athermal, the chemical free energy changes during quenching will only consist of the Gibbs energy difference between the parent and product phases. In this work, Equation (42) is used to calculate the thermodynamic driving force during martensitic transformation.

(42) $$f^{ch}\left(T\right)=G_{M}\left(T\right)-G_{A}\left(T\right)=\frac{T-T_{0}}{T_{0}}Q$$

where $T_{0}$ is the start cooling temperature; *Q* is the chemical-dependent relative latent heat between austenite and martensite.

The chemical concentration related critical energy (Equation (43)) introduced by Cool and Bhadeshia [33] is employed to calibrate *Q* in this quenching process simulation.

(43) $$\begin{array}{c} \Delta G_{crit}^{CB}=683+4009x_{C}^{0.5}+1980x_{Mn}^{0.5}+1868x_{Cr}^{0.5}, \end{array}$$

where $\Delta G_{crit}^{CB}$ is in J/mol and *x* is the mole fraction. Only the existing elements in the investigated steels are calculated in this equation.

Due to the large transformation Bain strains [34], the elastic large deformation approach developed by Borukhovich et al. [8,9] is used for the mechanical solution. Additionally, austenite was simulated as a mechanical isotropic phase, and martensite was treated as an anisotropic tetragonal phase. The applied elastic coefficients are shown in Table 1.

Current views about martensite morphology in low carbon steels describe that a prior austenite grain is divided into packets according to different habit planes, and each packet is further subdivided into blocks and sub-blocks, which decompose into a group of laths with the same orientation. Such an orientation relationship between austenite and martensite in lath martensite is crystallographically expressed as Kurdjumov–Sachs (K-S) orientations.

**Figure 3:**
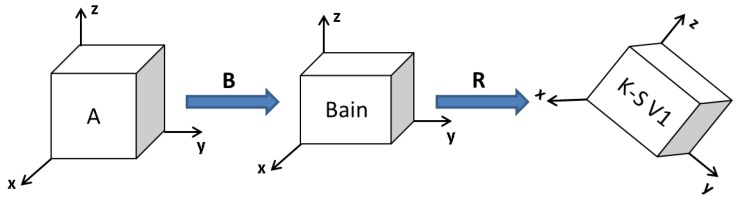
Schematic sketch of the generation of one Kurdjumov–Sachs (K-S) variant in the simulation.

In this work, austenite is taken as the reference phase. Therefore, martensite variants are obtained by first applying the Bain strains (**B**) to the austenite grain, then rotating according to the K-S orientation transformation matrices (**R**). Figure 3 briefly sketches the transformation sequence for one K-S variant. All 24 K-S orientation transformation matrices (**R**) are derived in paper [39].

**Figure 4:**
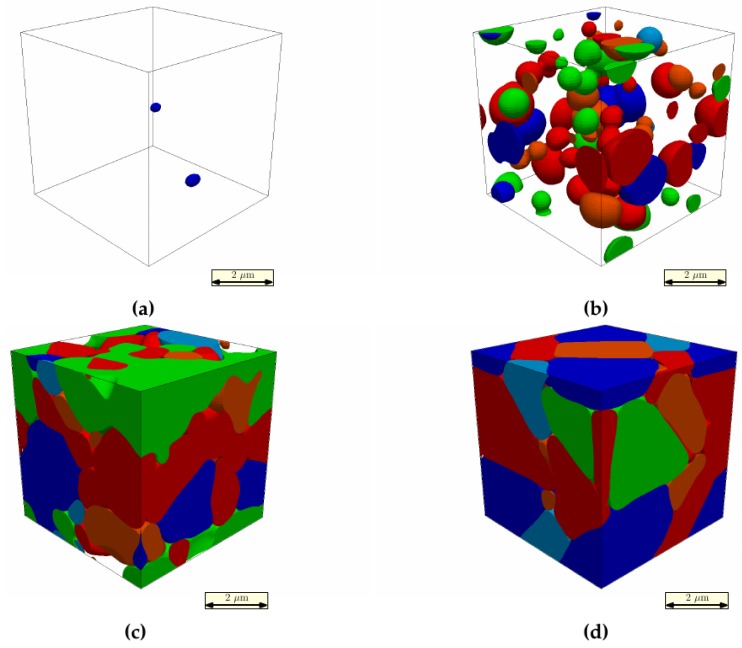
Martensite microstructure evolution of multiple K-S variants. Each color represents one K-S orientation variant. The same color with different shades is different K-S variants in the same Bain orientation. A single prior austenite grain; only one packet is expected due to the simulation domain limits. (**a**) After 3000 time steps; (**b**) after 6000 time steps; (**c**) after 9000 time steps; (**d**) after 20,000 time steps.

For the quenching microstructure simulation, a time step of 6 × 10^−4^ s was chosen. Since the size of the initial nucleation site is in the range of only a few nanometers, while the final martensite microstructure corresponds to several microns, the quenching simulation is performed with the grid spacing of 1 × 10^−8^ m and scaled up in the following simulation steps, in order to reduce the computational effort by approximately a factor of 10^3^. The interface width is set to five grid points, and the interfacial energy was set to 1.0 J/m^2^. Periodic boundary conditions were applied due to the use of the spectral mechanical equilibrium solver. The size of the computational domain was set to 64 × 64 × 64 grid points. Figure 4 shows the microstructure evolution of multiple K-S martensite variants at four different time steps (ts).

In general, a plate-like structure was obtained, which is shown in all martensite phase-field simulations with Bain orientations. Besides, more detailed microstructures in lower levels were first captured by our phase-field simulation. Through autocatalytic nucleation, the K-S variants of the same color with different shades (red and orange) grew together into blocks, and different blocks were further arranged into packets. Because of the calculation limitation on the box size, this example is not able to capture the full size of one packet in real martensite microstructure. Therefore, only the sub-blocks and blocks microstructures can be clearly revealed in Figure 4.

## 6. Results: Precipitation

During the tempering simulation with elevated temperatures at 1000 K and the specific material of 0.2 wt% carbon (0.9 at%), 1 wt% chromium and 2 wt% manganese, prolonged carbide nucleation and growth can be observed especially at grain boundaries with a total phase fraction of up to 4 vol% in the whole simulation domain.

**Figure 5:**
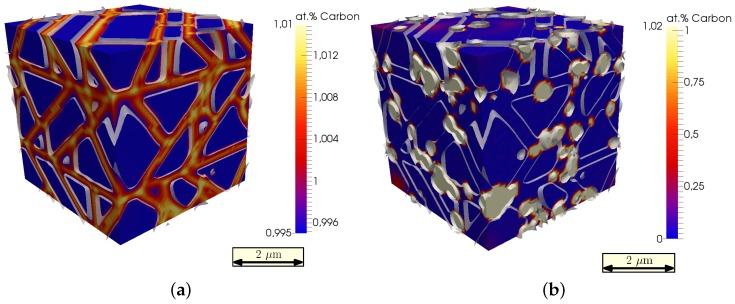
(**a**) Initial state and (**b**) final composition of carbon in the simulation box after 100,000 time steps.

In Figure 5a, the assumed initial composition is shown, as carbon tends to segregate into grain boundaries [13]. After 100,000 time steps, carbon is fully segregated into the newly-formed cementite precipitates, and nearly no carbon is left in the matrix, as can be seen in Figure 5b.

**Figure 6:**
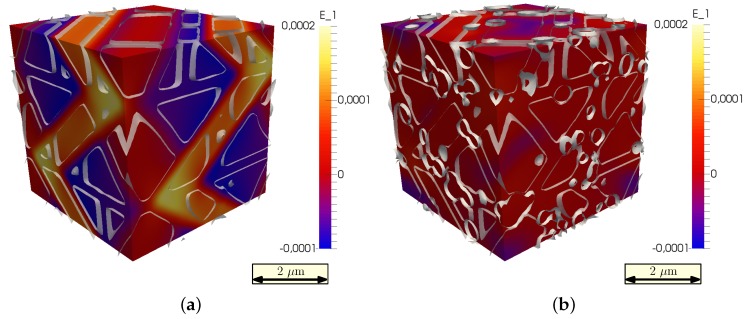
(**a**) Initial state and (**b**) final strain state of the simulation box after 100,000 time steps.

The transformation strain, which depends on the carbon composition, therefore decreases with a decreasing carbon fraction in the matrix. This can be seen by comparing the initial strain state in Figure 6a with the final state in Figure 6b. The final state of the simulated sample is less strained and therefore more ductile.

**Figure 7:**
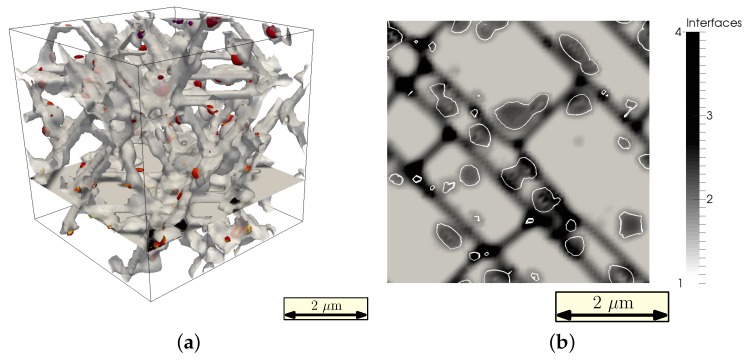
(**a**) Cutaway of the bulk area to only highlight the arrangement of $M_{3}C$ carbides and the grain boundaries; (**b**) 2D section through a 3D simulation box. Grain boundaries are dark grey, cementite grains are encircled with a white line.

Nucleation occurs mainly at grain boundaries, where the initial carbon composition is highest, and a greater driving force for carbide nucleation and growth exists. This can be seen in Figure 7a where the precipitates’ networks are clearly connected by the higher order grain boundaries. In the cut through the simulation box in Figure 7b, the black grain boundary regions are saturated with a concentration of cementites, shown as white circles.

This matches previous estimations, that cementite nucleates as fine precipitates favorably around defects like grain boundaries.

## 7. Results: Mechanical Testing Simulation

In this section, the modeled material sample resulting from the numerical procedure described in the two previous sections is subjected to a tensile testing calculation. Therefore, an elongation of 10% along the y-axis is applied, combined with a compression in the x and z direction, chosen such that the total deformation is volume conserving. The calculation domain is set to be ${\left(64\times 100\right)}^{3}$ nm, consistent with the previous calculations. The time step is chosen as $dt=1\times 10^{-5}$ s. The elastic coefficients of martensite and cementite are chosen according to Table 1. For the eigenstrain, a residual carbon composition of the martensite is assumed to be 0.14 at%, resulting in $\epsilon_{xx}^{*}=-0.00007217,\;\epsilon_{yy}^{*}=-0.00007217,\;\epsilon_{zz}^{*}=0.00043456$. The plasticity parameters are set as ${\dot{\gamma }}_{0}=1.0$ and k=10. The damage parameters are $p_{min}=0.06$ and $p_{max}=0.065$ accordingly.

**Figure 8:**
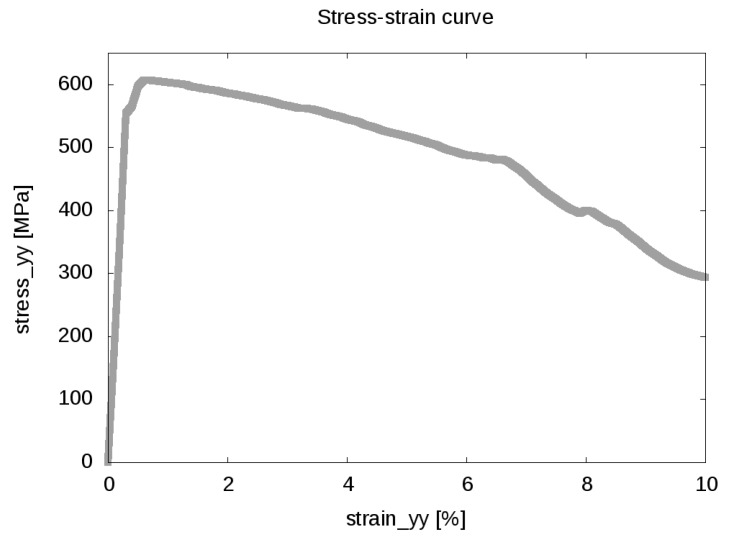
Stress-strain curve recorded during the tensile test simulation. The curve shows the yy-component of the average stress tensor during the increase of applied strain in the y-direction.

A characteristic stress-strain curve obtained during the mechanical testing is shown in Figure 8. As can be seen, the elastic regime lasts for less than half percent stretch, followed by the plasticity-governed deformation. Because of the relatively low plastic exponent (k=10), viscous behavior is observable leading to stress relaxation after the maximal stress of approximately $\sigma_{yy}\approx 600$ MPa. At stretch values over 6%, the damaged regions become significant for the material sample, and the stress relaxation increases dramatically. This is in good agreement with the chosen parameters of the damage model determining the damage to happen in the strain range between 6% and 6.5%. The discrete stress drops during the testing simulation are caused by the discrete box resizing steps. With the used box dimension of 64 in each direction, resizing can only occur in steps of slightly less than 2%. Using a larger box size is expected to minimize this undesired numerically effect. However, the values of maximal reached stress and the stretch intervals at which the plastic behavior and the damage control regime start are not influenced.

**Figure 9:**
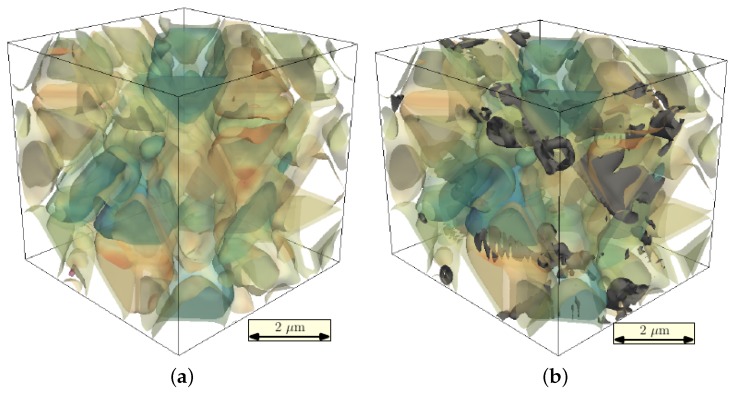
(**a**) Not deformed and (**b**) deformed microstructure. The colored surfaces mark the individual martensite grains. The final state corresponds to 10% stretch in the y-direction. The grey surfaces denote the maximally-damaged regions.

In Figure 9, the initial and the predicted final microstructure state of the simulated tensile test can be seen. Due to the complex morphology, the structure of the damaged regions is not obvious. However, in the final state, the damage variable reaches the maximal value of D=0.99 (due to the numerical limitations of the used spectral elastic solver, it is impossible to set D=1.0, since this would lead to zero stiffness tensor), which corresponds to total local failure of the material.

**Figure 10:**
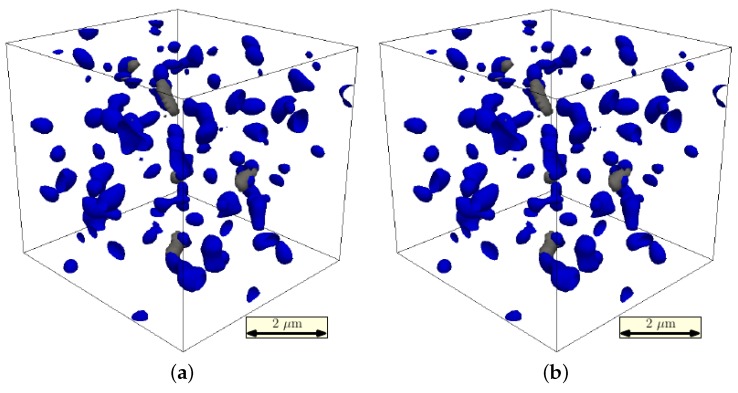
(**a**) Early damage state. The blue surfaces denote the carbides; the grey surface shows the initially damaged regions (D=0.05); (**b**) Final damage state. The grey surfaces denote the D=0.6 contour. The highest damage value reached in this state is D=0.99.

**Figure 11:**
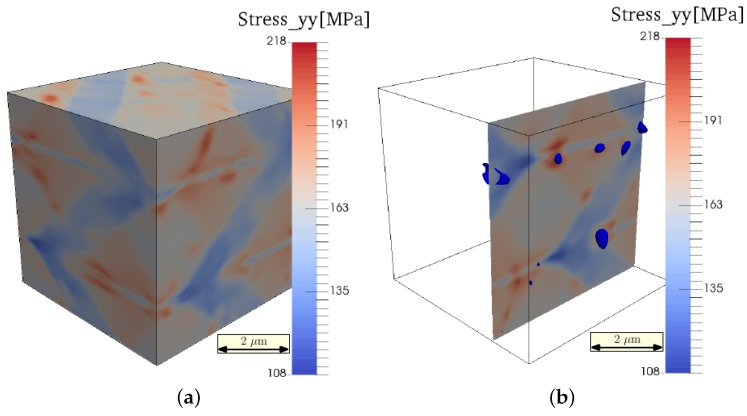
(**a**) Stress state after 1% stretch ($\sigma_{yy}$); (**b**) stress at a cut surface normal to the y-direction. The blue surfaces denote the precipitates at this plane.

In order to obtain a more detailed view of the damage process, in Figure 10, the initial damage nucleation and the final damage morphology are related to the carbides’ distribution. The first damage occurs in regions next to, or even between, the carbides. Once nucleated, the damage spreads from these positions along the martensite grains. This process can be understood, since the carbides lead to stress localization, as shown in Figure 11.

Thus, we can conclude, that due to the stress localization resulting from the presence of cementite precipitates, the initial damage formation occurs earlier at distinct regions, saving the rest of the material from extreme stresses by the dissipation of mechanical energy. Hence, the martensite regions experience moderate stress that is distributed almost homogeneously along each martensite grain (Figure 11). However, once the stress in these regions reaches the yield level and individual martensite grains are strained up to the damage initialization strain, the stress decay of the total system accelerates, as can be seen in the high strain region of the stress-strain curve (Figure 8).

## 8. Conclusions

In this work, steel processing steps from quenching resulting in martensite formation, over tempering accompanied by the formation of carbides to mechanical testing and the final material failure have been modeled. All simulations have been performed with the same software framework OpenPhase and could have been performed within one simulation run. This fact is owed to the generic free energy-based approach followed by the phase-field modeling tool chosen and expanded by the authors. In a scale-bridging approach, atomistically-informed materials models are integrated.

Considering the 24 different orientation variants together with a mechanical driving force term, the typical martensite microstructure has been successfully reproduced. Even though the spatial and temporal resolution prevents the simulation from yielding the exact picture of the quenching procedure at each calculation step, the model converges to the physically-correct microstructure, as can be seen on the grain boundary angles. In the end, however, this is the most important characteristic of the result, allowing further studies on the modeled material system.

While the martensite formation occurs at a time scale small enough to neglect diffusion processes, the carbon redistribution and especially carbon segregation to the grain boundaries is crucial during the tempering step. The effect can easily be observed, when monitoring the precipitation growth, which mainly occurs along the carbon-enriched grain boundaries. Additionally to the purely chemically-driven diffusion, also stress and strain effects are considered following the recently-formulated approach from [12]. However, this effect is less pronounced.

Finally, the mechanical behavior of the resulting material is studied in a mechanical testing simulation. The material is uniaxially-loaded, and the stress response is recorded. Additionally to the plasticity model, a damage evolution is considered in this part of the modeling process. Analysis of the damage distribution provides the insight that, though the carbides formed during the tempering step control the initial position of the damaged regions via stress localization, the final damage morphology follows the martensitic grains. This multi-scale approach can be applied to design optimal microstructures dependent on processing and materials composition.

## Figures and Tables

**Table 1:** Elastic coefficients.

**Elastic Coefficients of Austenite (500 °C) [35,36]**
$C_{11}=C_{22}=C_{33}=237\;GPa$
$C_{12}=C_{13}=C_{23}=117\;GPa$
$C_{44}=C_{55}=C_{66}=60\;GPa$
**Elastic Coefficients of Martensite (0 K)** [37]
$\begin{array}{cc} C_{11}=C_{22}=237\;GPa & C_{33}=258\;GPa \end{array}$
$C_{12}=C_{13}=C_{23}=144\;GPa$
$C_{44}=C_{55}=C_{66}=114\;GPa$
**Elastic Coefficients of Cementite (0 K)** [38]
$\begin{array}{cc} C_{11}=390\;GPa & C_{22}=345\;GPa \end{array}$
$C_{12}=C_{13}=C_{23}=160\;GPa$
$\begin{array}{cc} C_{33}=320\;GPa & C_{44}=20\;GPa \end{array}$
$C_{55}=C_{66}=135\;GPa$
